# Avian Toxins and Poisoning Mechanisms

**DOI:** 10.1007/s13181-022-00891-6

**Published:** 2022-04-26

**Authors:** Kara A. Yeung, Peter R. Chai, Brendan L. Russell, Timothy B. Erickson

**Affiliations:** 1 Harvard Affiliated Emergency Medicine Residency (HAEMR) Program, Mass General Brigham, Boston, MA USA; 2 Department of Emergency Medicine, Division of Medical Toxicology, Mass General Brigham, Vining St. Neville House Boston, Boston, MA 02115 USA; 3 The Fenway Institute, Boston, MA USA; 4 The Koch Institute for Integrated Cancer Research, Massachusetts Institute of Technology, Cambridge, MA USA; 5 Division of Psychosocial Oncology and Palliative Care, Dana Farber Cancer Institute, Boston, MA USA; 6 Harvard Humanitarian Institute, Cambridge, MA USA

**Keywords:** Poisonous birds, Avian, Ornithology, Toxinology, Toxic chemical defense

## Abstract

All around the world, there are species of birds that have developed the ability to acquire toxic chemicals in their bodies making them less palatable or even lethal when consumed or contacted. Exposure to poisonous bird species is rare among humans, yet their poisons can produce serious clinical outcomes. In this study, we conducted a literature search focusing on seven avian species: the pitohuis (*Pitohui* spp.), blue-capped ifrita (*Ifrita kowaldi)*, European quail (*Cortunix corturnix coturnix*), spur or spoor-winged goose (*Plectropterus gambensis*), North American ruffed grouse (*Bonasa umbellus*), Brush bronzewings (*Phaps elegans*), and European hoopoes and woodhoopoes (*Upupa epops* and *Phoeniculus purpureus*, respectively). We present the geographic distribution of each poisonous bird, toxin physiology and origin, clinical signs and symptoms of poisoning, cases of human toxicity if available and discuss the birds’ ability to prevent self-intoxication. Our results suggest that most cases of contact with toxic birds produce mild symptoms as most of these birds apart from the European quail (*C. c. corturnix*) and North American ruffed grouse (*B. umbellus*) are not commonly consumed by humans. Furthermore, we discuss several methods of toxin acquisition in these bird species, which are mostly diet acquired apart from the hoopoes and woodhoopoes (*Upupa* and *Phoeniculus* spp.) who have a symbiotic relationship with chemical-producing bacteria in their uropygial glands. In summary, our study provides a comprehensive review of the toxic physiology, clinical manifestations, and evolutionary insight to avian toxins.

“*It is not only fine feathers that make fine birds.” -Aesop*

## Introduction

Birds have evolved to acquire a variety of adaptations to survive the hostile animal kingdom. While there are those who use camouflage to hide in their environment or developed increased agility to fly or run away from predators, others have developed ability to acquire toxic chemicals in their bodies making them less palatable or even lethal when consumed. Some birds have additionally developed symbiotic relationships with other organisms for mutual survival and chemical defense against predators. While poisonous animals abound in the animal kingdom, human exposures to avian species are rare yet their poisons can produce serious clinical outcomes.

Bird species that possess chemical defense by containing or using behaviorally one or more chemical substances to deter predators or parasites have often been described as poisonous or toxic to humans and animals alike [1, 2]. Common poisonous avian species will be discussed in this comprehensive review article which include the Pitohuis (*Pitohui* spp*.*, *Melanorectes nigrescens, Ornorectes cristatus,* and *Pseudorectes ferrugineus*), blue-capped ifrita (*Ifrita kowaldi)*, European quail (*Cortunix corturnix coturnix*), spur or spoor-winged goose (*Plectropterus gambensis*), North American ruffed grouse (*Bonasa umbellus*), Brush bronzewings (*Phaps elegans*), European hoopoes and woodhoopoes (*Upupa epops* and *Phoeniculus purpureus*, respectively). In this manuscript, common poisonous bird species will be highlighted with emphasis on the toxicologic properties of these compounds. Specific treatments beyond supportive care for poisonings will also be discussed. Furthermore, theories behind the evolutionary ability of these unique species to prevent self-intoxication will also be explored.

## Methods

We conducted a literature search in MEDLINE / PubMed, Hollis, and Google Scholar of the English language, which included peer-reviewed investigations as well as case reports describing toxicological profiles, pharmacology, and pathophysiology on the avian species of interest. The search terms included each avian species: (*Pitohui* spp. which include hooded pitohui (*Pitohui dichrous*)*,* variable pitohui (*Pitohui kirhocephalus*)*,* black pitohui (*Melanorectes nigrescens*), crested pitohui (*Ornorectes cristatus*), rusty pitohui (*Pseudorectes ferrugineus*), and white-bellied pitohui (*Pitohui icnertus*), blue-capped ifrita (*Ifrita kowaldi*), European migratory quail (*Coturnix coturnix coturnix*), Spur or Spoor-Winged goose (*Plectropterus gambensis*), North American ruffed grouse (*Bonasa umbellus*), Bronzewings (*Phaps elegans, Phaps chalcoptera*), European hoopoes (*Upupa epops*), and Green woodhoopoes (*Phoeniculus purpureus*)); the avian species’ toxin of interest: (homobatrachotoxin, palasonin, cantharidin, demethylcantharidin, grayanotoxin, and monofluoroacetate); and other keywords listed after each specific species: (toxin, toxicity, poison, chemical defense). We recognize that literature and research on toxic or poisonous avian species are limited and thus included both peer-reviewed and non-peer-reviewed manuscripts and literature. Exclusion criteria included gray literature, lay press, letters to the editor, and editorials. We excluded articles that had no discussion of toxicological poisoning related to the birds of interest or discussion of clinical presentations related to poisonings or toxic exposure. There was no time restriction or specific date range placed on our search or selection process.

Chemical structures were created with the program ACD/ChemSketch. High-resolution photographs of each bird species were selected from the Macaulay Library at the Cornell Lab of Ornithology used with permission.

## Results and Data Summary

**Map 1:**
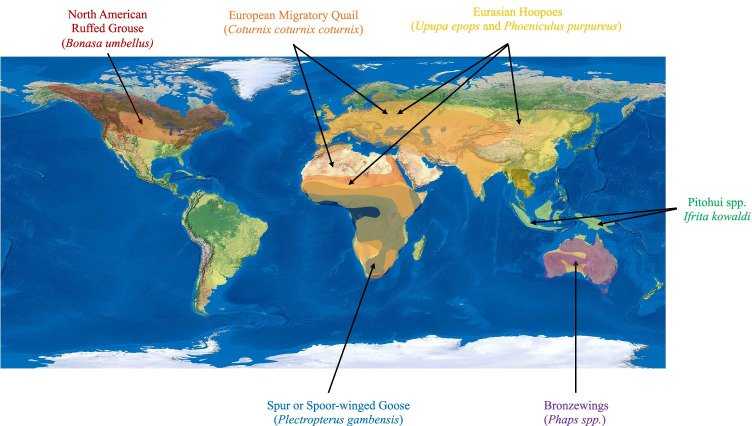
Geographic distribution of poisonous bird species

Table 1 summarizes each poisonous bird’s toxin physiology and origin, along with clinical signs and symptoms of toxicity. Table 2 provides the chemical structure of each avian toxin. Map 1 depicts the geographic distribution of these poisonous bird species.

**Table 1.** The avian species’ geographic location, season of poisoning, toxin physiology and origin, and clinical signs and symptoms are summarized below

Species	Location/Season of Poisoning	Toxin/Physiology	Toxin Origin	Clinical Signs and Symptoms
Pitohui spp. *Ifrita kowaldi*	New Guinea	Batrachotoxin and homobatrachotoxin Voltage gated sodium channel inhibitor	Diet of Melrid beetles (*Choresine* spp.)	Convulsions, muscle contractions, numbness, dyspnea, salivation
European migratory quail (*Coturnix coturnix coturnix*)	Human poisoning observed in Europe, Africa during Autumn season	Coniine Nicotinic acetylcholine receptor agonist	Diet of *Conium maculatum, Hyoscyamus niger, Solanum nigrum, Oenanthe crocata, Galeopsis ladanum*	Acute rhabdomyolysis (coturnism), myalgias, muscle stiffness, weakness, cramps, myoglobinuria
Spur or Spoor-Winged goose (*Plectropterus gambensis*)	Sub-Saharan Africa	Palasonin – cantharidin and demethylcantharidin Inhibits phosphatase 2A and receptors	Diet of blister beetles (*Meloidae* spp*.*)	Dermatitis and blisters, vasoconstriction, dehydration, increased contractility
North American Ruffed grouse (*Bonasa umbellus*)	Human poisoning observed in Northeastern USA, East Canada, UK during late winter, early spring	Grayanotoxin Voltage-sensitive sodium channel activation	Diet of *Kalmia latifolia*	Dizziness, weakness, nausea, vomiting, paresthesia, arrhythmias
Bronzewings (*Phaps* spp.)	Australia	Monofluoroacetate converted to fluorocitrate (active toxin) Disrupts cellular energy metabolism – inhibition of phosphofructokinase	Diet of *Acacia, Gastrolobium* spp*., Oxylobium* spp.	Convulsions, excessive salivation, vomiting, defecation, tenesmus, ECG changes
European hoopoes (*Upupa epops*) Green woodhoopoes (*Phoeniculus purpureus*)	Africa, Asia, Europe	Volatile compounds Symbiotic bacteria in uropygial glands	Symbiotic relationship with bacteria in glands	No evidence for systemic symptoms Foul smelling odor when inhaled

**Table 2:**
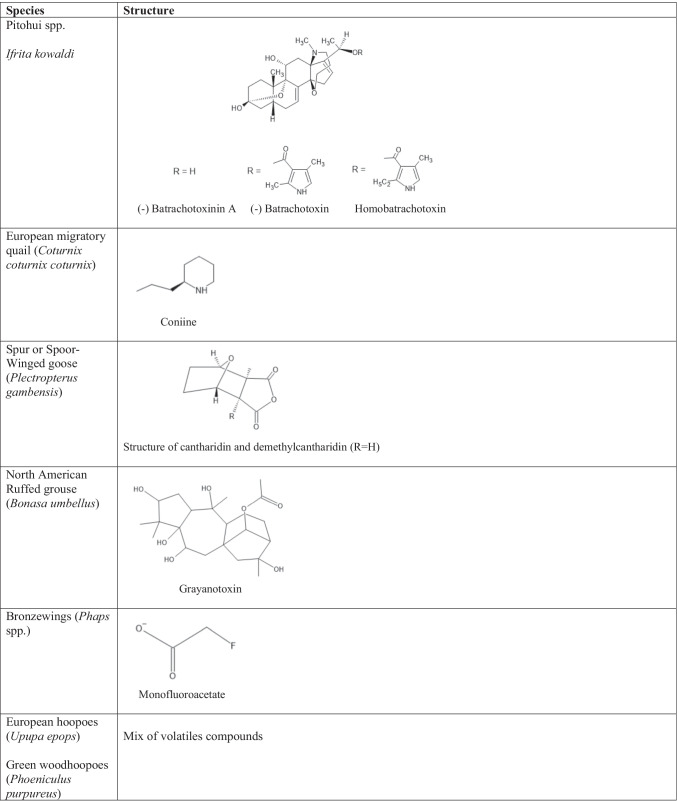
The names and chemical structures of each toxin of interest are illustrated in the table below

## Discussion of Poisonous Bird Species

### Pitohuis and Ifrita kowaldi

**Figure 1.**
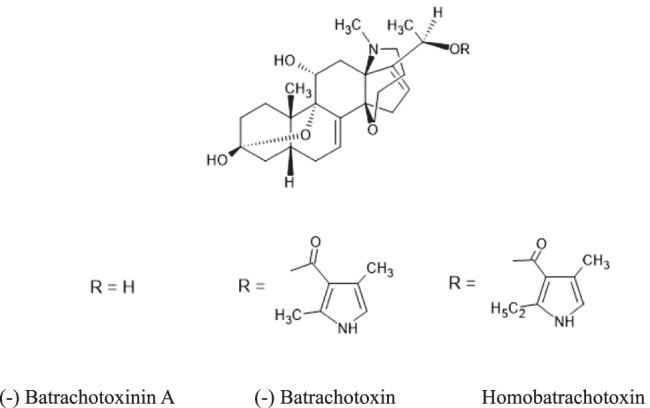
Batrachotoxin Structures adapted from Ligabue-Braun, Rodrigo, and Carlini, Célia Regina. “Poisonous Birds: A Timely Review. ”*Toxicon (Oxford)*, vol. 99, 2015, pp. 102–108

The Pitohuis and *Ifrita kowaldi* are colorful birds that are endemic to New Guinea. There are six species of Pitohuis with varying levels of toxicity with the hooded (*Pitohui dichrous*) and variable pitohui (*Pitohui kirhocephalus*) more toxic than other related species [3]. The hooded pitohui can be identified with its distinct jet-black head and brick red belly (Photograph 1). Black (*Melanorectes nigrescens*) and crested pitohui (*Ornorectes cristatus*) have traces of toxicity and the rusty (*Pseudorectes ferrugineus*) and white-bellied pitohui (*Pitohui icnertus*) have no toxicity. The blue-capped ifrita (*Ifrita kowaldi*) is also a bird endemic to New Guinea restricted to high montane rainforests (> 1,500 m). Species plumage is yellowish brown with a blue and black crown (Photograph 2). Although their toxicity profile is similar to the Pitohuis*, I. kowaldi* belongs to a different family [4]. Initial analysis of the skin and feathers from the birds revealed a single, toxic alkaloid called homobatrachotoxin (Fig. 1) [5]. Subsequent studies have shown that the Pitohuis and *I. kowaldi* contain a series of batrachotoxins [3]. These toxins are concentrated in the breast and belly feathers with the thought that the toxins not only deter predators from consuming the bird itself but can be transferred to nests and eggs, thereby deterring egg-eating predators [3]. Furthermore, the presence of toxins in the feathers has been proposed to promote defense against ectoparasites [6].

Batrachotoxins are potent neurotoxic steroid alkaloids with neuro- and cardiotoxic properties [7, 8]. These toxins were originally found in the skin of neotropical frogs from the genus *Phyllobates* commonly known as poison-dart frogs (family Dendrobatidae) [9]. These toxins occur at high levels in the true poison dart frogs from Columbia (*Phyllubetes terribilis)* and to a lesser degree in other Central American species [7]. They target voltage-gated sodium channels at receptor site II in nerve and muscle membranes and stabilize the open forms of these channels [10]. Binding of the receptors causes persistent activation of the channel subsequently leading to depolarization of cells. While no antidote exists, certain agents can be used to reverse membrane depolarization. For example, tetrodotoxin can be used through its antagonistic effect on sodium channels [11]. In rodent models, batrachotoxins are some of the most potent alkaloids known. The intravenous LD_50_ in mice is 2 µg/kg for batrachotoxin and 3 µg/kg for homobatrachotoxin [10, 12]. Meanwhile, its derivative, batrachotoxinin A, has a much lower toxicity with an LD_50_ of 1 µg/kg. Symptoms manifested in rodent studies include muscle contractions, convulsions, salivation, dyspnea, and death following lethal (LD_50_) doses.

While contact with dendrobatid frogs can cause severe symptoms, toxin exposures from the birds are milder. The most likely explanation is that birds carry lower levels of toxin compared to dendrobatid frogs [5]. Symptoms of exposure include numbness, burning, nausea, and bitter taste if consumed [13]. These toxins are nonvolatile, but if released into the air from dander or feather bits, they can be inhaled or cause upper respiratory irritation [3]. Researchers who worked directly with these bird species experienced sneezing, numbness and burning of oral mucosa [5]. Natives of New Guinea report that these species have long been avoided for consumption unless prepared in a specific manner as they are known for having a bitter odor and sour taste [14].

There are various hypotheses as to how the *Pitohui* and *I*. *kowaldi*. acquired their toxicity. Dumbacher et al. proposed that while both Pitohuis and *I. kowaldi* demonstrated toxicity, they were found in different geographic regions of New Guinea and occupied different niches, and yet, both were poisonous [3]. Furthermore, the Pitohuis had varying levels of toxicity with the hooded (*P. dichrous*) being most toxic and white-bellied pitohui (*P. icnertus*) having no toxicity further supporting that toxicity was acquired through environment and most likely through diet [3]. It is proposed that melrid beetles (*Choresine* spp*.*) may be a source of batrachotoxins as they are observed to be part of the diet of *Pitohui* and *I*. *kowaldi* [15]. In addition, melrid beetles are also found in Colombian rainforests, which could link the similarities in toxicity with these birds and poisonous dendrobatid frogs despite being indigenous to different continents. An alternative theory is that both birds and beetles acquire toxins through a plant source either through ingestion of plant seeds or from insects who obtain molecular scaffolds for batrachotoxin after eating plants. However, *I. kowaldi* is almost exclusively insectivorous, whereas the Pitohuis are omnivorous making this plant theory less likely [15]. Phylogenetic comparisons have shown that the clusters of both the Pitohuis and *I. kowaldi* appear at the tips of the phylogeny, but overall, there is a higher rate of losing the poisonous trait opposed to gaining, suggesting that many lineages have subsequently lost that toxic ability [16].

### Common Quail (*Coturnix coturnix coturnix*)

The common quail (*Coturnix coturnix coturnix*) is a small and compact bird (16–18 cm in length; wingspan 32–35 cm) with streaks of brown with white eye stripes (Photograph 3). As this species of quail is migratory, they have long wings compared to the short-winged gamebird species [17]. Toxicity is primarily associated with the European subspecies of migratory quails and observed around autumn season during quail migratory seasons [18, 19].

Consumption of these birds can cause a toxic myopathy associated with acute rhabdomyolysis termed *coturnism*. Symptoms include weakness, myalgias, muscle stiffness, and cramps. Laboratory abnormalities include myoglobinuria, increased levels of aldolase, aspartate transaminase, creatine kinase, and lactate dehydrogenase. Treatment is generally supportive with crystalloid fluid replacement, urine alkalinization, and hemodialysis for severe cases of renal failure. Plasmapheresis may be an option for life-threatening cases of rhabdomyolysis [20].

There have been several case reports of patients developing rhabdomyolysis after consumption of quail meat with mild-to-moderate symptoms and laboratory abnormalities requiring supportive care and urine alkalinization [21–23]. In one case report by Gokhan et al., a 58-year-old male presented to the emergency department in Turkey with complaints of weakness, muscle pain, nausea, vomiting, and decreased and dark urine [22]. The patient had consumed quail meat approximately four hours prior to symptoms onset. Laboratory workup was notable for elevated lactate dehydrogenase (LDH) 872 IU/L (reference 120–130), creatine phosphokinase (CPK) 17,480 IU/L (25–190), aspartate aminotransferase (AST) 834 IU/L (10–40), alanine aminotransferase (ALT) 376 IU/L (10–40), and urinalysis showed myoglobinuria and proteinuria. Patient’s medical history excluded any other possible cause of rhabdomyolysis, and thus, his presentation was consistent with acute rhabdomyolysis from quail meat. He was admitted to the hospital and received intravenous fluids, mannitol, and urine alkalinization with intravenous sodium bicarbonate and orally administered acetazolamide. His muscular pain and weakness resolved in three days and muscle enzymes normalized over nine days, and he was ultimately discharged. While this case report did not comment on time of year, three other case reports have reported that patients diagnosed with coturnism all consumed quail during the autumn migratory season [21].

**Figure 2.**
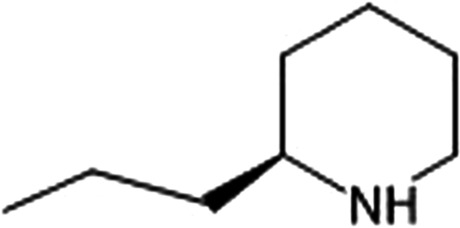
Coniine

The toxicity is theorized to be from an alkaloid toxin, coniine which is commonly found in poison hemlock (*Conium maculatum)*. Coniine is a nicotinic acetylcholine receptor (nAChR) agonist (Fig. 2). Studies have proposed that the toxin is acquired through the bird’s consumption of seeds from poison or spotted hemlock (*Conium maculatum*), hemlock water dropwort (*Oenanthe crocata*), red hemp-nettle (*Galeopsis ladanum*), as well as the anticholinergic alkaloid-containing plants henbane (*Hyoscyamus niger*) and black nightshade (*Solanum nigrum*) [24, 25]. Other alkaloids such as stachydrine (L-proline betaine) have been studied but have not been shown to be toxic. A focused study on the red-hemp nettle (*Galeopsis ladanum)* and the compound stachydrine demonstrated that feeding both the seeds extracts or quail meat to mice did not produce signs or symptoms or rhabdomyolysis [26]. An alternative hypothesis to coturnism observed in humans is a combined effect of the bird toxin and a hereditary enzyme deficiency in the affected individual; however, no specific enzyme has yet been identified [27].

### Spur or Spoor-winged Goose (*Plectropterus gambensis*)

**Figure 3.**
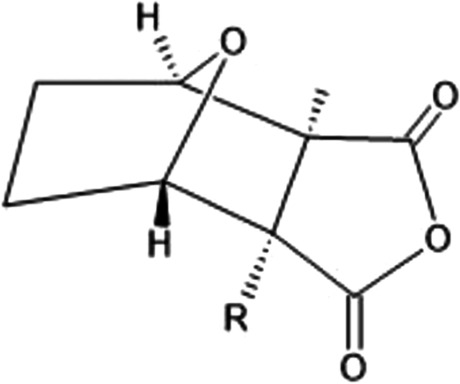
Structure of Cantharidin and demethylcantharidin (R = H)

The Spur (or Spoor)-winged goose (*Plectropterus gambensis*) is found in wetlands throughout sub-Saharan Africa. These birds are mainly black with a white face and white wing patches (Photograph 4). The geese acquire the toxin from consumption of blister beetles (family Meloidae) [28]. The main compounds in the beetles are terpenes, specifically, cantharidin and demethylcantharidin, commonly known as palasonin [29] (Fig. 3). Cantharidin binds to protein phosphatase 2A and inhibits serine-/threonine-specific protein phosphatases [30], which are important in reversible protein phosphorylation processes. These processes are involved in various cellular functions including neurotransmission, muscle contraction, glycogen synthesis, T-cell activation, and cell proliferation [31–33].

Two routes of exposure to cantharidin can occur. The toxin can be absorbed directly through skin and mucous membranes. Common skin manifestations include a dermatitis rash with blister formation. Specifically, cantharidin causes release of serine proteases that cause desmosomal plaque disruption leading to acantholysis, intradermal blistering, and nonspecific lysis of the skin [34]. The toxic effects on mucosal membranes can lead to blistering in the oropharynx, dysphagia, and abdominal cramping [35–37]. Furthermore, poisoning after oral ingestion can cause dehydration due to excess free fluid losses. This occurs through inhibition of renal cortical collecting ducts, increase contractility, vasoconstriction, endothelial cell leakage, and overall pro-inflammatory state from upregulated cytokine genes [38–40]. Systemic symptoms include abdominal pain, hematuria, cool, mottled extremities, and dehydration. In rare cases, priapism has been described, a potential desired effect of commercially available cantharidin or “Spanish Fly” aphrodisiac [40, 41]. Furthermore, cantharidin can cause spontaneous abortions in females and has historically been used as an abortifacient [42, 43]. Mortality has been commonly observed in farm animals and in some cases humans who consume the actual beetle [44–46]. However, there is no recent literature or research that has documented or reported human toxicity from cantharidin after consumption of this avian species. There are no specific antidotes to treat cantharidin poisoning. Supportive care and administration of oral activated charcoal for recent ingestions are recommended.

### North American ruffed grouse (*Bonasa umbellus*)

**Figure 4.**
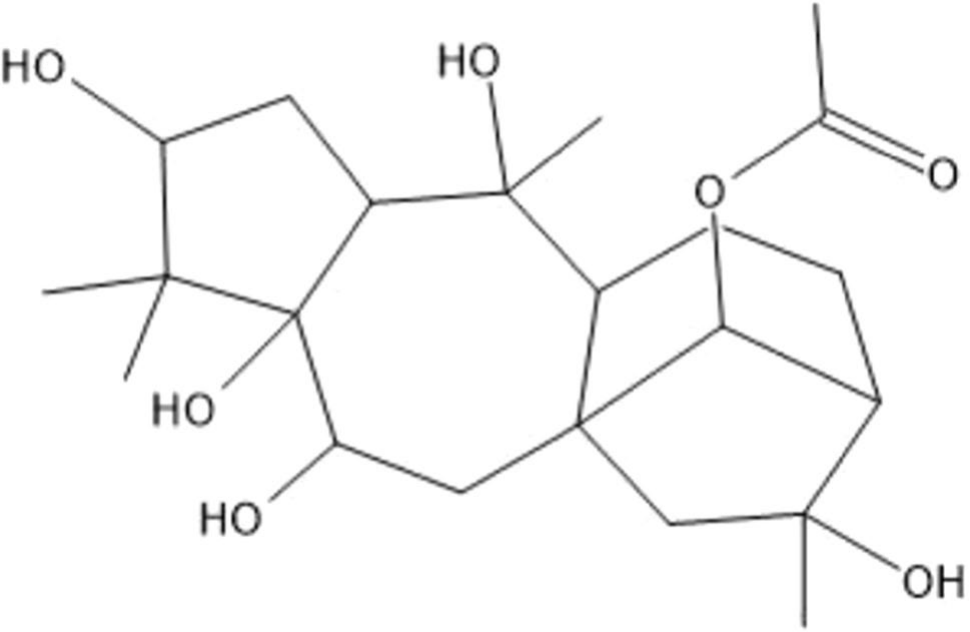
Grayanotoxin

The North American ruffed grouse (*Bonasa umbellus*) is a non-migratory bird found in forests of the US Appalachian Mountains across Canada to Alaska. They are chunky, medium-sized birds that appear in both grey and brown morphs with ruffs that appear on the side of their necks (Photograph 5). They harbor the toxin, grayanotoxin, which is acquired through consumption of the mountain laurel (*Kalmia latifolia*) (Fig. 4). Reports of human poisoning from grouse consumption have occurred during late winter and early spring as it is thought that the snow-covered terrain forced the birds to seek food in trees and tall shrubs. Specifically, the leaves and buds of the laurel would be consumed during that season [47]. Grayanotoxin is a diterpene that can bind to group II receptor sites in cellular voltage-gated sodium channels leading to prevention of inactivation of these channels, thus keeping the cell in a depolarized or “open” state [48]. This can lead to both neurotoxic and cardiotoxic effects. Symptoms of systemic toxicity include dizziness, weakness, diaphoresis, hypersalivation, nausea, vomiting, and paresthesia. Severe toxicity can lead to life-threatening arrhythmias due to the increase in resting sodium permeability and activation of voltage-sensitive sodium channels [49]. Management is mainly supportive care, and if necessary, atropine can be administered for symptomatic bradycardia.

Grayanotoxins have largely been studied in honey containing *Rhododendron* spp. nectar. Human toxicity, often referred to as “mad honey disease” [50], occurs after ingestion of contaminated honey, more commonly observed along the Black Sea coast of Anatolia. Poisonings from grouse consumption have been documented as early as the late eighteenth to mid-nineteenth century when physicians observed side effects in those who had consumed grouse meat [47, 51]. While there are no recent published case reports of human poisoning in the twentieth and twenty-first century, medical literature from 1821 to 1882 has shown human poisonings after grouse meat consumption in multiple cities from northeastern USA, eastern Canada as well as the UK where they receive shipments of grouse from the USA and Canada [51–53]. Dr. Jacob Bigelow described ten cases of human poisoning from grouse meat consumption observed on the northeastern coast of the USA. In one case, he described a 60-year-old man who consumed grouse meat one hour prior to developing symptoms of gastrointestinal upset, dizziness, weakness, nausea, and vomiting. He received ipecac and fluids but remained delirious for several hours prior to resolution of his symptoms [54]. Per case reports from Dr. Bigelow’s reports, no fatalities have been documented as symptoms have been mostly mild and required supportive care only.

### Bronzewings (*Phaps elegans*, *Phaps chalcoptera*)

**Figure 5.**
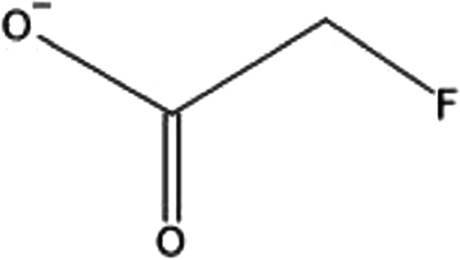
Monofluoroacetate

The Bronzewings (*Phaps elegans* and *Phaps chalcoptera*) are medium-sized pigeons that are native to Australia. While each species has slightly distinct plumage, all bronzewing pigeons share the characteristic patches of red, blue, and green on their wings (Photograph 6). They are known to acquire monofluoroacetate from consumption of flowering plant species such as wattle or acacia (*Acacia* spp.)*, Gastrolobium* spp., and shaggy pea (*Oxylobium* spp.) [2, 55]. Sodium monofluoroacetate is also the main component of the potent rodenticide commercially known as Compound 1080 [56] (Fig. 5).

**Figure 6.**
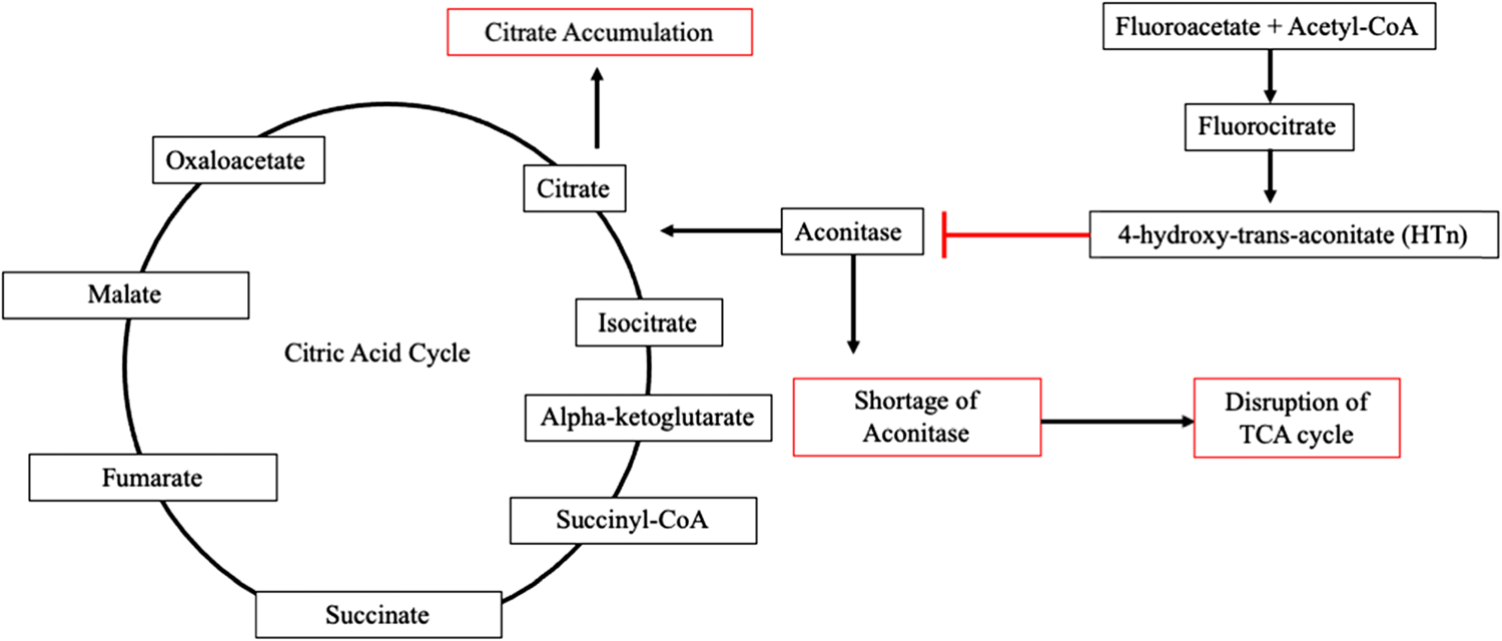
Fluorocitrate is converted to 4-hydroxy-trans-aconitate (HTn), which leads to inactivation of aconitase, thus leading to citrate accumulation and shortage of aconitase leading to disruption of citric acid (TCA) cycle

Monofluoroacetate is both neuro and cardiotoxic. It can be absorbed in both the respiratory and gastrointestinal tract as well as mucous membranes. Fluoroacetate’s mechanism of action affects cellular respiration, in the citric acid or Krebs cycle [57]. Once absorbed, fluoroacetate is combined with acetyl CoA and metabolized to fluorocitrate. While citrate can continue through the citric acid cycle, fluorocitrate does not. Fluorocitrate is converted to 4-hydroxy-trans-aconitate (HTn), which leads to inactivation of aconitase, thus inhibiting citrate and succinate metabolism within the citric acid cycle. High citrate concentrations can lead to inhibition of phosphofructokinase, which leads to further disruption of cellular energy metabolism [58–60] (Fig. 6). Furthermore, fluoride can bind to calcium causing significant hypocalcemia. Studies in sheep have shown acute cardiac toxicity manifesting as both myocardial ischemia and arrhythmias [61]. Toxicity observed in canines include central nervous system excitation and gastrointestinal tract hypermotility [56]. There is no recent documentation of human poisoning from consuming bronzewing meat; most observations are made from carnivores and zoo animals that have consumed these birds [62, 63]. Signs and symptoms include seizures, excessive salivation, vomiting, defecation, and tenesmus [64, 65]. From veterinary data, treatment of Compound 1080 poisoning includes administering acetamide, but if not available, initiating a sodium bicarbonate infusion [66]. Fomepizole (4-methylpyrazole or 4-MP), the alcohol dehydrogenase inhibitor used to treat methanol and ethylene glycol poisoning, has been shown in one rodent study to reduce toxic effects of a similar compound to Compound 1080 via reduction of oxaloacetate production, which then reduces erythrofluorocitrate production [67]. While it is possible that 4-MP may be used to treat human poisoning due to known mechanism of action, there are no known recent studies that have investigated its use on sodium monofluoroacetate toxicity, and therefore, initial management remains supportive with decontamination Figs. 7,8, 9, 10, 11, 12, 13 and 14.

**Photograph 1:**
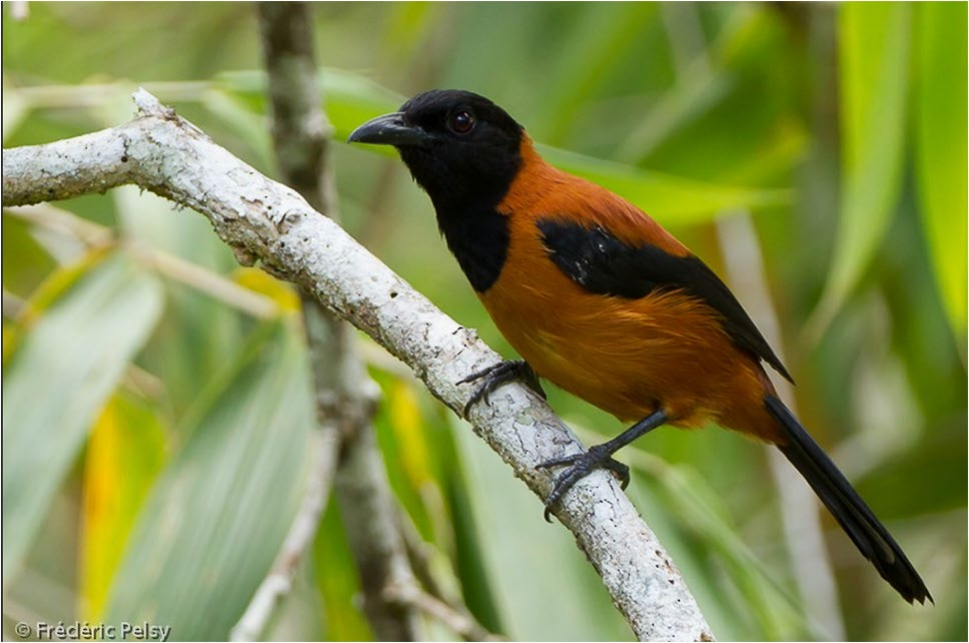
Hooded pitohui (*Pitohui dichrous)* Frédéric PELSY / Macaulay Library at the Cornell Lab (ML206167861)

**Photograph 2:**
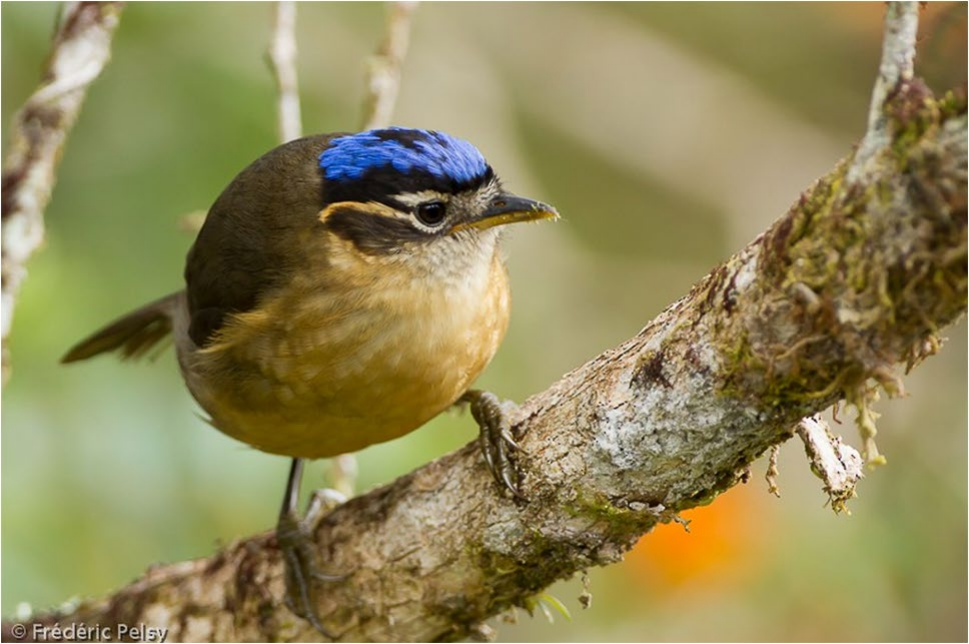
Blue-capped ifrita (*Ifrita kowaldi)* Frédéric PELSY / Macaulay Library at the Cornell Lab (ML206205861)

**Photograph 3:**
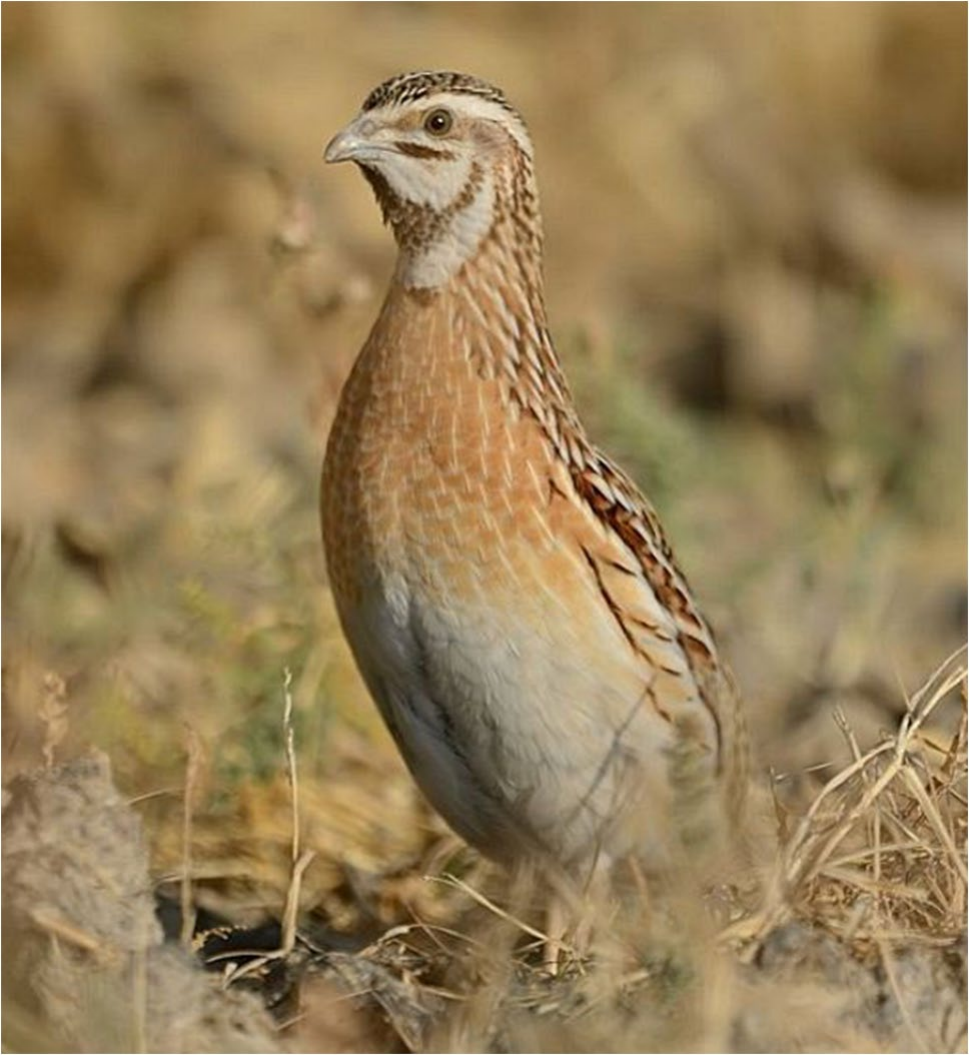
Common quail (*Coturnix coturnix) European subspecies (Coturnix coturnix coturnix)* Rajesh Shah / Macaulay Library at the Cornell Lab (ML379431801)

**Photograph 4:**
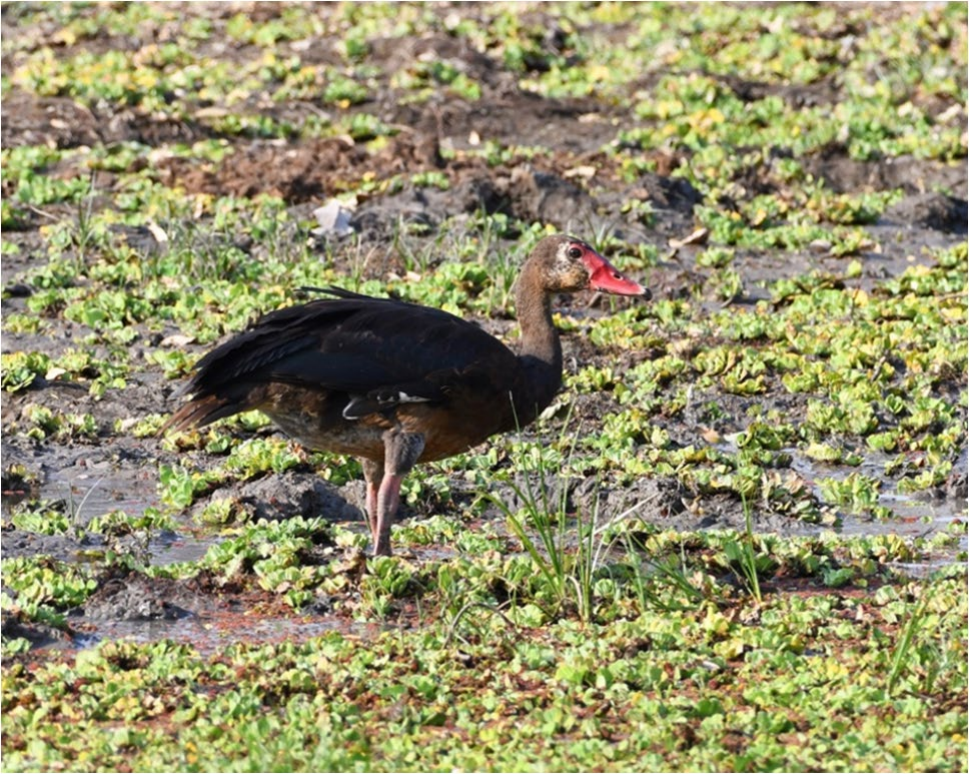
Spur (or Spoor)-winged goose (*Plectropterus gambensis*) Greg Hudson / Macaulay Library at the Cornell Lab (ML375975451)

**Photograph 5:**
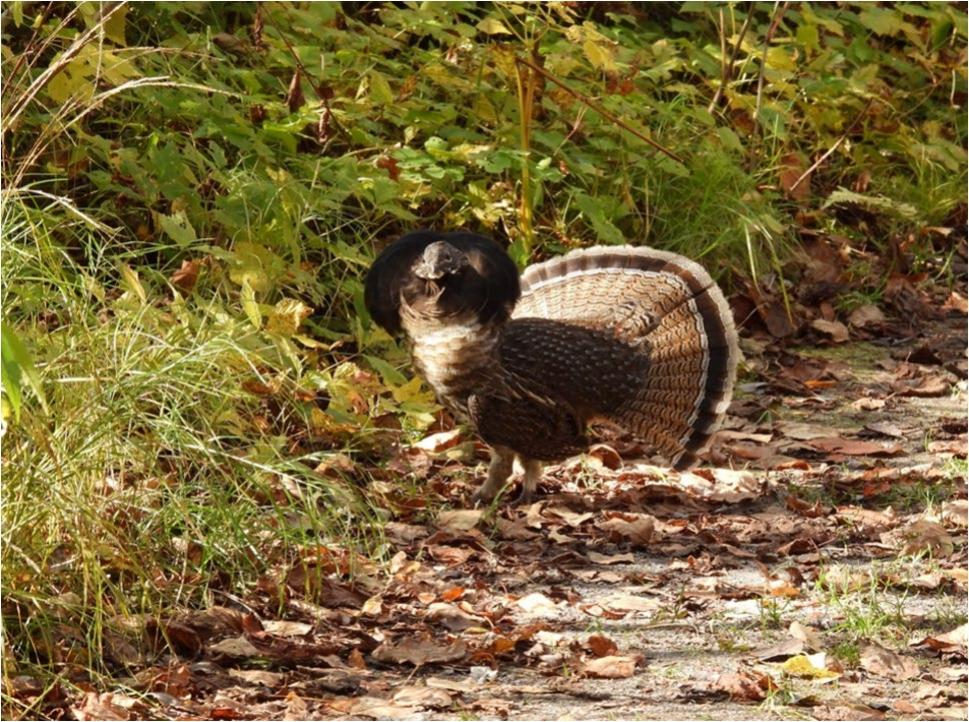
North American ruffed grouse (*Bonasa umbellus)* Rejean Beauchesne / Macaulay Library at the Cornell Lab (ML380801351)

**Photograph 6:**
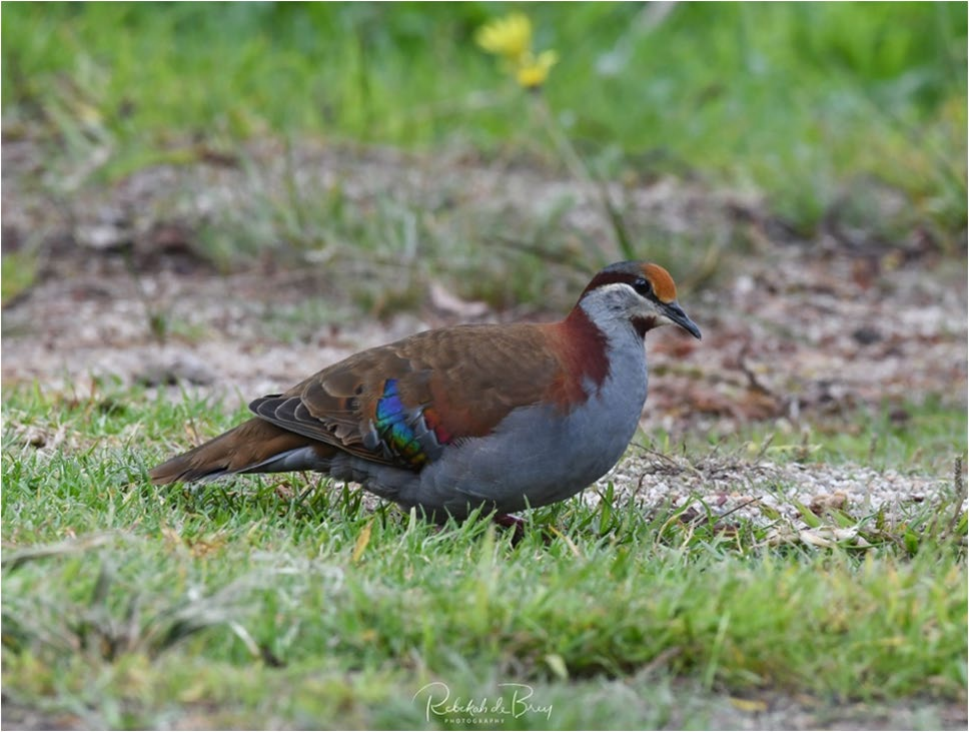
Bronzewings *(Phaps elegans)* Rebekah de brey / Macaulay Library at the Cornell Lab (ML378586341)

**Photograph 7:**
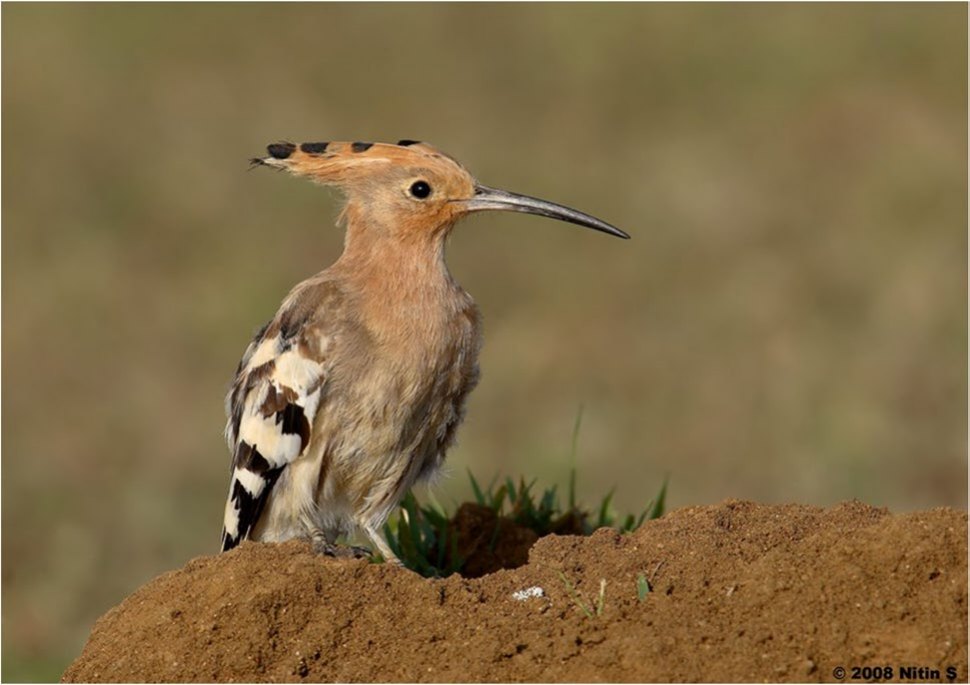
European hoopoes (*Upupa epops*) Nitin Srinivasa Murthy / Macaulay Library at the Cornell Lab (ML380618991)

### Hoopoes

The European hoopoes (*Upupa epops*) and green woodhoopoes (*Phoeniculus purpureus*) are found in Africa, Asia, and Europe and have distinct “crowns” of feathers (Photograph 7). Uropygial glands are specialized exocrine glands in avian species that produce a range of biochemicals. Hoopoes have symbiotic bacteria in their uropygial glands, which produce noxious volatile compounds such as dimethyl sulfide [68]. Martin-Vivaldi et al. [69] demonstrated that common symbionts in the European hoopoes’ uropygial glands are *Enterococcus* spp*.* but hypothesize there could be other bacterial species that were not cultivable with standard methods. The composition of the uropygial secretions in European hoopoes does change between breeding and non-breeding seasons. Breeding females and nestlings will produce malodorous dark secretions that contain anti-microbial properties. During the non-breeding season, a white secretion is produced, which does not have volatile chemicals [69]. There are no known major human toxicities from Hoopoes as they are not known to be consumed by humans. Researchers of these birds have reported smelling the noxious fumes for several hours when they get the chemicals on their hands after handling the birds [68].

## Ability to Withhold Toxin

Many theories have been proposed as to how birds have acquired such toxins. The intriguing evolutionary question is how birds acquire and harbor such toxins in their bodies without getting poisoned themselves. Ecologists and chemists alike have attempted to study and elucidate the idea of sequestered defensive chemicals (SDCs) [70]. While species such as the European hoopoes and woodhoopoes can avoid self-intoxication given they have a dedicated gland that contains the chemicals, species such as the *Pitohui* spp. and *Ifrita* contain toxin in their tissues and feathers.

Like poison dart frogs, these birds do not succumb to their own lethal doses of batrachotoxin contained within their skin and tissues. In a recent study, a single amino acid substitution on the poison dart frog's sodium channel rendered resistance to the effects of batrachotoxin [71]. Studies of natricine snakes (*Thamnophis* spp.) demonstrate that they have mutations in their sodium channel proteins allowing them to be resistant to the effects of tetrodotoxin after consuming poisonous newts [72, 73]. It is plausible that both predator and prey have adapted by genetically altering their proteins, thus enabling them not only to be resistant to toxins sequestered within their body but also to toxins consumed.

Other explanations beyond genetic mutations include herbivores having the ability to modify ingested toxic alkaloids from plants in the gut into non-toxic bases. It is proposed that re-activation of non-toxic alkaloids into their toxic forms is conducted through cytochrome P450 enzymes [74]. Another example is the chrysomelid beetle avoiding self-intoxication by moving toxic bases effectively to specialized exocrine glands and away from susceptible tissues [75]. While these are all proposed theories for birds as demonstrated by other vertebrate and invertebrate species, further investigation will need to be performed to identify the exact chemical and biological adaptations for each poisonous bird species. While there is no clear explanation as to how avian species sequester and maintain non-toxic forms in their body, it can be speculated based on observations from non-avian species that self-intoxication could be prevented through metabolic pathways, specialized organs for sequestration, or genetic modifications that confers resistance.

## Conclusion

The toxicologic risk from exposure to various poisonous birds lies on a spectrum. Most often, dermal exposure leads to mild symptoms, while ingestion leads to systemic and even lethal toxicity. While most reports of human toxicity have been observed in both quail (*C. c. coturnix*) and the North American ruffed grouse (*B. umbellus*), the risk of human toxicity from the other avian species is extremely low based on the limited to no reports of human exposure or toxicity.

Poisonous birds have evolved multiple strategies to use chemicals as defense against predators and to protect their broods. From dietary sources to symbiotic relationship with bacteria, these bird species have developed sophisticated methods of using chemical defense. Globally, poisonous birds alike have evolved to use their environment as part of their defense strategies. Apart from the hoopoes and woodhoopoes, most poisonous birds acquire their toxins secondarily through diet of either plant or invertebrate species.

Most intriguing is the ability of birds to manage sequestered defensive chemicals and not succumb to self-intoxication. While there have been studies on other vertebrate and invertebrate species to investigate this protection, the direct mechanism of protection, whether it be modification of toxins to non-toxic bases during storage or nucleotide polymorphisms in targeted receptors in birds has yet to be elucidated fully. Further studies defining the intricacies of avian chemical defense and their potential clinical applications are warranted.
